# A machine learning model for predicting postoperative complication risk in young and middle-aged patients with femoral neck fractures

**DOI:** 10.3389/fsurg.2025.1591671

**Published:** 2025-08-26

**Authors:** Yixin Huang, Dongze Lin, Bin Chen, Xiaole Jiang, Shanglin Shangguan, Fengfei Lin

**Affiliations:** ^1^ Department of Orthopedics, Fuzhou Second General Hospital, Fujian Provincial Clinical Medical Research Center for Trauma Orthopedics Emergency and Rehabilitation, Fuzhou, China; ^2^ Fujian University of Traditional Chinese Medicine, Fuzhou, Fujian, China; ^3^ Anxi County Hospital, Quanzhou, Fujian, China

**Keywords:** femoral neck fracture, internal fixation failure, risk factors, machine learning, prediction model

## Abstract

**Objective:**

Femoral neck fractures are the most common type of hip fracture, and the postoperative complications associated with these fractures significantly affect patients’ quality of life and healthcare costs. The objective of this study was to develop a predictive model using machine learning (ML) techniques to assess the risk of postoperative complications in young and middle-aged patients with femoral neck fractures.

**Methods:**

We retrospectively analyzed data from 899 young and middle-aged patients with femoral neck fractures who underwent surgical treatment between September 2019 and June 2024. Key predictors affecting postoperative complications were identified through LASSO regression and multifactorial logistic regression analyses. Several machine learning (ML) models were then integrated for comparative analysis. Ultimately, the best-performing model was selected, and its interpretation was provided using SHAP values to offer a personalized risk assessment.

**Results:**

The study results indicate that intraoperative reduction quality, medial cortex comminution, fracture types, posterior tilt angle, early postoperative weight-bearing, and removal of internal fixation devices are significant predictors of postoperative complications. The logistic regression model demonstrated the best performance on the test set, with an area under the curve (AUC) of 0.906, accuracy of 0.877, sensitivity of 0.748, and specificity of 0.903. Additionally, SHAP analysis identified the seven most important features in the model, providing clinicians with an intuitive tool for risk assessment.

**Conclusions:**

This study successfully developed and validated a logistic regression-based predictive model, augmented with SHAP explanations, providing an effective tool for assessing the risk of postoperative complications in young and middle-aged patients with femoral neck fractures.

## Introduction

Hip fractures are reported to occur more than 1.7 million times globally each year (1), with femoral neck fractures accounting for 50% of all hip fractures (2, 3), making them the third most common type of fracture in traumatology (4). Among these patients, 2%–11% are young individuals (5), typically due to high-energy trauma. Although various surgical treatment methods are available for these fractures, multiple meta-analyses have shown that the incidence of complications has not significantly changed (6–8). These complications can result in a substantial decrease in patients' health-related quality of life, prolonged hospital stays, increased readmission rates, and higher medical costs (9, 10).

Previous studies have shown that the probability of complications following internal fixation of femoral neck fractures increases with the complexity of the injury, with severely displaced fractures (Garden III, IV) closely associated with an increased risk of postoperative complications (11–13), loss of intraoperative reduction quality (14, 15), and comminution of the medial cortex (11). Other potential risk factors include smoking, excessive alcohol consumption (16), and comorbid medical conditions (17). Although these are well-known general risk factors, surgeons still face challenges in translating them into individual risk assessments for specific patients. If the specific risks of postoperative complications after femoral neck fractures (i.e., risk stratification) could be more precisely predicted, it would assist surgeons in identifying and closely monitoring those patients at high risk of complications.

At present, to our knowledge, there is a lack of predictive models with sufficient and long-term follow-up sample sizes for postoperative complications of femoral neck fractures, which can be used to calculate the probability of postoperative complications for patients in order to stratify between high-risk and low-risk patients. Machine learning methods (Machine Learning, ML) have attracted attention, as they possess a more advanced ability to predict patient outcomes compared to traditional methods. The advantages of ML include their ability to handle complex nonlinear relationships between predictors and produce more stable predictions, which may be one of the reasons why the development of these ML predictive models is becoming increasingly common in orthopedic surgery (18–20). Among existing models, for example, Zhu et al. developed a predictive model for early complications by combining deep learning with clinical data and x-ray images (21), and Wang et al. enhanced the understanding of the risk of postoperative femoral head necrosis after femoral neck fractures through machine learning models and multivariate analysis (22). These studies had insufficient sample sizes, and their predictive results have not been confirmed in subsequent prospective studies.

This study retrospectively analyzed data from young and middle-aged patients with femoral neck fractures who underwent surgical treatment at the Second General Hospital of Fuzhou between September 2019 and June 2024, with the aim of developing an initial machine learning (ML) predictive model to estimate the incidence of postoperative complications for individual patients following surgical treatment of femoral neck fractures.

## Method

### Guideline

This study adhered to the Transparent Reporting of a Multivariable Prediction Model for Individual Prognosis or Diagnosis (TRIPOD) statement guidelines for reporting prognostic studies (23) and was approved by the Ethics Committee of the Second Hospital of Fuzhou (approval number: 2025008). In accordance with the Declaration of Helsinki, revised in 2013, the study anonymized the data. Due to its retrospective nature, no additional harm was inflicted on participants, and thus informed consent was not required.

### Sampling and sample size calculation

Machine learning models often involve a large number of hyperparameters and require substantial sample sizes. Currently, there is no specialized method for calculating the sample size specifically tailored to machine learning predictive models. In the context of sample size calculation for predictive models, the 10 EPV (events per variable) rule is a widely adopted empirical guideline. This rule suggests that for model development based on binary outcomes or survival events, at least 10 outcome events should be available for each predictor variable (i.e., regression coefficient *β* value) to ensure the accuracy and reliability of the model's sample size. In this study, we aim to construct a clinical prediction model for the risk of postoperative complications following femoral neck fracture surgery, with 26 predictor variables planned for inclusion. This implies that a minimum sample size of 260 is required to meet the 10 EPV criterion.

### Study population

This retrospective follow-up study was conducted on patients who underwent internal fixation for femoral neck fractures with at least three months of follow-up. The study population consisted of patients who were discharged from the Second General Hospital of Fuzhou after receiving internal fixation for femoral neck fractures between September 2019 and June 2024. A total of 28 clinical characteristics of 899 femoral neck fracture patients were collected through methods such as searching the inpatient electronic medical record system, medical imaging information system, laboratory information system, and follow-up. After applying the inclusion and exclusion criteria, the study ultimately included 899 patients with femoral neck fractures.

Inclusion criteria: (1) Patients diagnosed with femoral neck fractures through imaging and treated with internal fixation, aged between 18 and 75 years; (2) Femoral neck fracture patients with complete baseline data; (3) Follow-up time of at least three months; (4) No moderate to severe pain or limited mobility in the affected hip joint before the fracture.

Exclusion criteria: (1) Pathological or bilateral fractures; (2) A history of long-term use of corticosteroids; (3) Patients who have experienced an acute myocardial infarction, cerebrovascular accident, severe trauma, or major surgery within the past six months; (4) Preoperative conditions affecting hip joint function, such as developmental dysplasia of the hip; (5) Surgery performed more than two weeks after the injury; (6) Patients lost to follow-up during the follow-up process.

### Primary outcome measures

**Figure 1:**
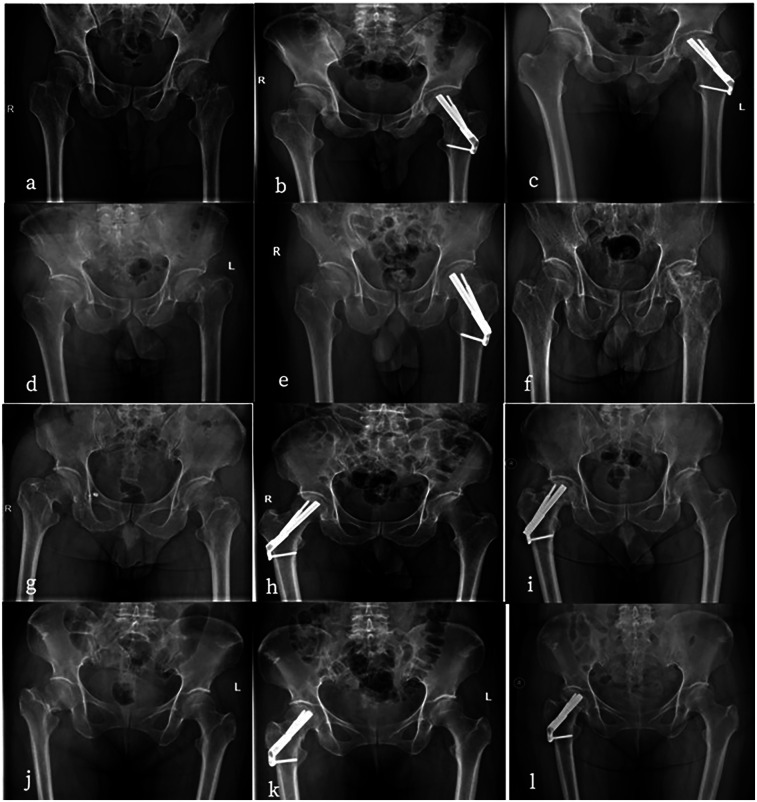
**(a–c)** Are all 56-year-old males. a has a preoperative anteroposterior hip radiograph showing a left femoral neck fracture (Garden IV type) with severe displacement. **(b)** Is a postoperative anteroposterior radiograph after FNS surgery. **(c)** developed femoral head necrosis two years after surgery. **(d–f)** Are all 35-year-old males.d has a preoperative anteroposterior radiograph of the hip showing a left femoral neck fracture (Garden IV type). **(e)** Is a postoperative anteroposterior radiograph after FNS surgery. **(f)** developed femoral head necrosis two years after the removal of the FNS internal fixation. **(g–i)** Are all 48-year-old females. (g's) preoperative anteroposterior hip radiograph shows a left femoral neck fracture (Garden IV type). **(h)** Is in a near-anatomic reduction position postoperatively. **(i)** Began weight-bearing two weeks after surgery and developed femoral head necrosis six months after follow-up. **(j–l)** Are all 36-year-old females. (j's) preoperative anteroposterior radiograph of the hip shows a right femoral neck fracture (Garden IV type). **(k)** Is a postoperative anteroposterior radiograph after FNS surgery with poor reduction quality. **(l)** Developed femoral head necrosis one year after surgery.

The outcome of interest predicted by the ML algorithm is the probability of postoperative complications in young and middle-aged patients with femoral neck fractures. Complications mainly include femoral neck shortening, femoral head necrosis, nonunion, internal fixation device complications, heterotopic ossification, infection, and poor wound healing, among others (Figure 1).

Femoral Neck Severe Shortening (>10 mm): The Zlowodzki method is used to assess the degree of femoral neck shortening (24). This method involves mirroring the healthy side to the affected side on a double hip anteroposterior x-ray to establish a baseline, then measuring the shortening in the horizontal (*X*-axis) and vertical (*Y*-axis) directions. Using the angle *θ* between the *Y*-axis and the femoral neck axis, the axial shortening distance *Z* is calculated using the formula *Z* = *Y*sin(*θ*) + *X*cos(*θ*). Based on the *Z* value, the degree of shortening is categorized as mild (<5 mm), moderate (5–10 mm), and severe (>10 mm), with >10 mm considered severe shortening.

Femoral Head Necrosis: This study assesses traumatic femoral head necrosis according to the 2019 revised ARCO staging system (25). The staging is as follows: Stage I—MRI shows linear low signal; Stage II—x-ray or CT shows sclerosis or osteoporosis of the femoral head; Stage III is divided into IIIA (collapse <2 mm) and IIIB (collapse >2 mm), showing subchondral fractures or flattening of the femoral head; Stage IV—x-ray shows joint space narrowing, indicating late-stage osteoarthritis.

Nonunion: According to the diagnostic criteria of the U.S. Food and Drug Administration (FDA) (26), poor fracture healing or nonunion can be diagnosed based on the following symptoms: pain and tenderness at the fracture site, and x-ray films showing a clear fracture line without callus formation. If there are no signs of healing at 9 months postoperatively and no healing trend in the last 3 months, it indicates poor healing.

Internal Fixation Device Complications: This includes internal fixation cutout and internal fixation failure. Internal fixation cutout (26) refers to the situation where the internal fixation structure remains intact, but due to displacement at the bone-implant interface, the device protrudes outward and causes pain. Internal fixation failure (27) refers to the failure of internal fixation due to loosening, breakage, or other reasons, which affects fracture healing.

Heterotopic Ossification: Referring to the diagnostic criteria of Brooker for heterotopic ossification (28), x-ray or CT examination shows signs of abnormal calcification or bone islands around the hip.

Infection (29): The diagnosis of osteomyelitis requires a comprehensive consideration of clinical symptoms, laboratory tests, and imaging assessments. Specifically, clinical symptoms include postoperative fever, local redness, and discharge; laboratory tests focus on abnormalities in white blood cell counts, erythrocyte sedimentation rate, and C-reactive protein levels; imaging-wise, x-ray examination is used to observe bone destruction and new bone formation, MRI aids in early diagnosis and determining the extent of infection, and PET/CT is used to assess patients with metal implants. Ultimately, pathological examination remains the gold standard for confirming infection.

### Surgical methods

All surgeries were performed under general anesthesia with patients positioned in the semi-lithotomy position. A team of experienced orthopedic surgeons, each with a minimum of five years of trauma surgery training, conducted the procedures. The surgical team comprised three senior surgeons and two fellows. The specific surgeon for each procedure was selected based on the patient's condition and the availability of the team. The reduction method employed for femoral neck fractures involved closed reduction. If the outcome of the closed reduction was not satisfactory, open reduction was subsequently performed.

#### FNS

The patient was placed in the supine position. After successful anesthesia, closed reduction of the fracture was performed under the guidance of a C-arm x-ray machine. After satisfactory reduction, a 3 cm incision was made below the greater trochanter. A guide pin was inserted into the subchondral bone of the femoral head, and the required FNS length was measured. The main FNS nail was inserted along the guide pin, and the distal locking screw was tightened. After confirming satisfactory fracture reduction and internal fixation position under the C-arm x-ray machine, the incision was closed.

#### FNS + ARS

Based on the surgical procedure of the FNS group, an ARS screw was inserted parallel and superior to the main FNS nail, with the screw tip located in the subchondral bone of the femoral head.

#### FNS + MSP

A medial plate was placed in the medial femoral neck according to the surgical procedure in the FNS group.

#### MCS

The patient was placed in the supine position. After successful anesthesia, closed reduction of the fracture was performed under the guidance of a C-arm x-ray machine. After satisfactory reduction, a 5 cm incision was made below the greater trochanter, and three parallel cancellous screws were inserted into the subchondral bone of the femoral head. After confirming satisfactory fracture reduction and internal fixation position under the C-arm x-ray machine, the incision was closed.

### Characteristic variable

Predictive factors include: (1) demographic information: gender, age, height, weight, BMI (Body Mass Index), CCI score (Charlson Comorbidity Index score) (30), alcohol abuse, and smoking; (2) fracture-related factors: injured side, intertrochanteric fracture (31A1.1 type), medial cortex comminution, Garden classification, Pauwels classification, and injury mechanism; (3) preoperative test indicators: Hb (hemoglobin), WBC (white blood cell count), PLT (platelet count), and D-dimer levels; (4) surgery-related parameters: surgery time, preoperative waiting time, removal of internal fixation devices, reduction quality (Gotfried) (31), reduction quality (Garden index) (32), intraoperative blood loss, reduction method (open or closed), and posterior tilt angle (Posterior_Tilt) (33); (5) surgical methods: Femoral Neck System (FNS), Femoral Neck System combined with Anti-Rotation Screw (FNS + ARS), Femoral Neck System combined with Medial Support Plate (FNS + MSP), and Cannulated Compression Screws (ccs); (6) postoperative characteristics: early postoperative weight-bearing and the final Harris score after surgery. The values and definitions of the variables can be found in Supplementary Appendix 1.

### Missing data

Variables with missing values include: early postoperative weight-bearing with 10 missing values (accounting for 2.48%), reduction quality with 7 missing (1.73%), fracture type with 8 missing (1.98%), removal of internal fixation devices with 4 missing (0.99%), CCI index with 13 missing (3.23%), and injury mechanism with 3 missing (0.74%). Missing data were imputed using the MissForest algorithm (34).

### Model development and validation

The entire dataset was randomly divided into a training set (70%) and a validation set (30%). Based on the training set, we developed a reference model and nine machine learning models to predict the probability of postoperative complications. In this study, we first used R.3.4.2 (glmnet 4.1.2) to perform Least Absolute Shrinkage and Selection Operator (LASSO) regression analysis for feature selection and to adjust the model's complexity. LASSO can shrink variable coefficients to prevent overfitting and address severe multicollinearity issue (35). Based on the results of LASSO, we further used SPSS.24 for multivariate logistic regression analysis to identify feature factors with *P*-values less than 0.05. Subsequently, we employed Python (Pysklearn 0.22.1) to randomly divide patients into training and testing sets in a 7:3 ratio using the random number method, with the training set containing 629 cases and the testing set containing 270 cases. We established a comprehensive analysis model with multiple classification models using Python (sklearn 0.22.1, xgboost 1.2.1, lightgbm 3.2.1), including (1) Extreme Gradient Boosting (XGBoost), (2) Logistic Regression, (3) Light Gradient Boosting Machine (LightGBM), (4) Random Forest, (5) Adaptive Boosting (AdBoost), (6) Decision Tree, (7) Support Vector Machine (SVM), (8) K-Nearest Neighbors (KNN), and (9) Gaussian Naive Bayes (GNB). We then trained and tested the aforementioned parameter models (repeated for 10 samples), analyzed the importance of training and testing set indicators in different models, and selected the optimal model.

To assess the predictive performance of the algorithms, the following performance metrics were used: (1) Model discrimination ability, (2) Calibration curve, (3) Clinical Decision Curve (Decision Curve Analysis, DCA), and (4) Recall curve (Precision-Recall curve). The discrimination ability of the model was assessed by calculating the area under the ROC curve (AUC). The AUC ranges from 0.5 to 1.0, where an AUC of 1.0 indicates perfect discrimination and 0.5 indicates no discrimination ability (36). Additionally, the calibration curve was used to assess the goodness of fit of the model, ensuring that the predicted probabilities match the actual outcomes (37). Clinical Decision Curve Analysis helps to evaluate the clinical applicability of the model at different thresholds, providing an assessment of net benefit at specific risk thresholds (38). Python (sklearn 0.22.1) was used to plot the Precision-Recall (PR) curve, which is widely used to evaluate the performance of models. The area under the PR curve provides a valuable supplement to existing model evaluation methods (39). Training, validation, and testing of the optimal model: The training set underwent 10-fold cross-validation and was evaluated with the test set. The predictive model with the best performance in both the derivation and validation cohorts was incorporated into an online prediction tool.

### Model interpretation

Python (shap 0.39.0) was used to plot SHAP values for explaining the importance and contribution of the model, and to interpret the model results by calculating the contribution of each feature to the prediction outcome. The SHAP (SHapley Additive exPlanations) method is a technique that ranks the importance of input features and explains the results of predictive models, addressing the “black box” issue (40). The SHAP method provides both global and local explanations for model interpretation. Global explanations offer consistent and accurate attribute values for each feature in the model, showing the association between input features and postoperative complications. Local explanations demonstrate specific predictions for individual patients by inputting specific data. Additionally, in this study, SHAP was constructed for individual samples (41).

### Statistical analysis

Continuous variables are expressed as the median and interquartile range (IQR), with comparisons made using the Mann–Whitney *U* test. Categorical variables are expressed as counts and percentages, and comparisons are made using the chi-square test. A two-sided *p*-value of less than 0.05 is considered statistically significant. All analyses were conducted using SPSS.24, R.Studio (3.4.2), and Python (3.4.3).

## Results

### Baseline data

**Figure 2:**
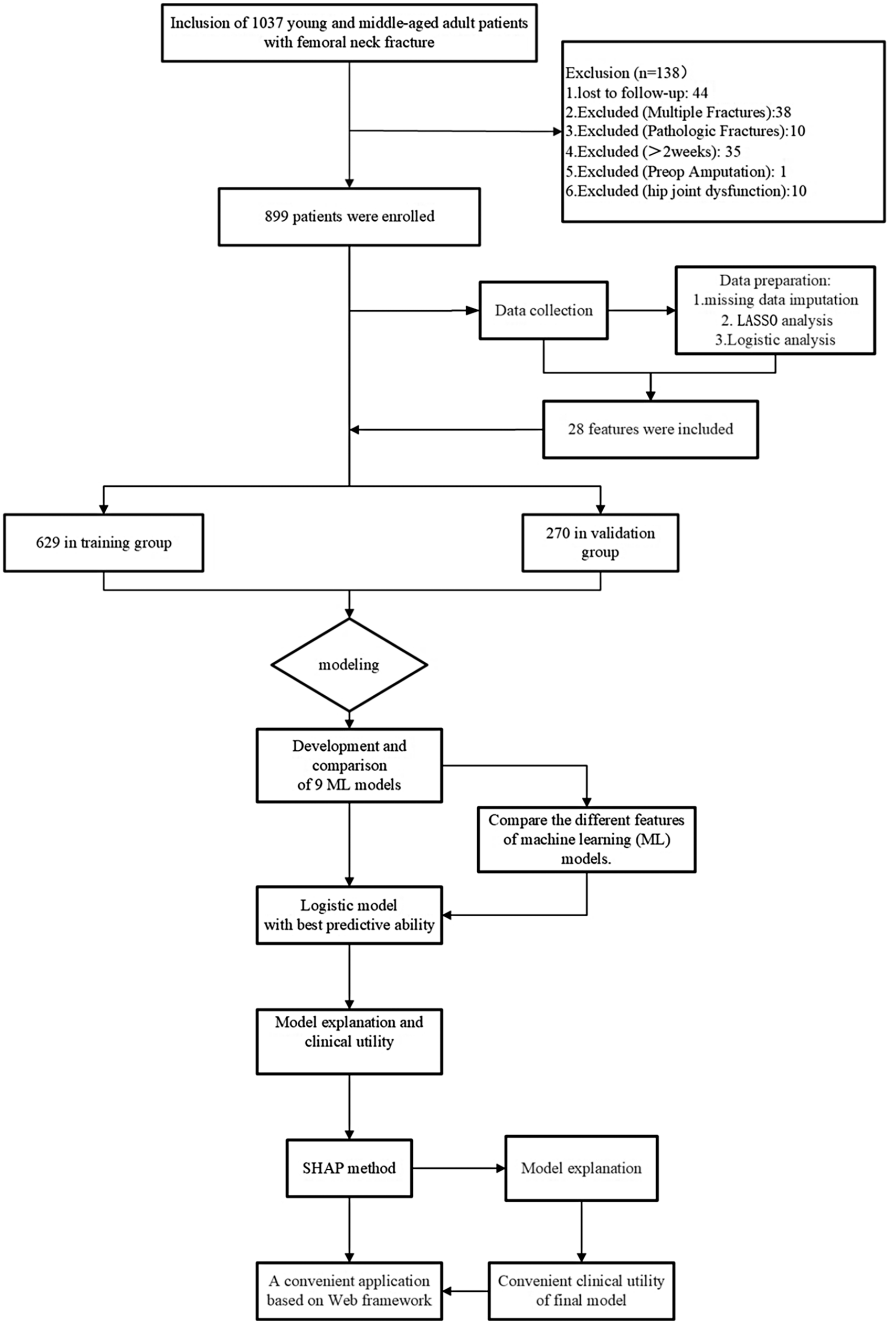
Flowchart.

This retrospective study aims to identify a predictive model for a cohort of 899 young and middle-aged patients with femoral neck fractures. During the study period, 38 patients with multiple fractures, 10 with pathological fractures, 35 with fractures older than 2 weeks, 10 with preoperative hip joint dysfunction, 1 who had previously undergone amputation surgery, and 44 with severe missing data were excluded. The remaining 899 femoral neck fracture patients were allocated to separate training and validation groups in a 7:3 ratio. Details of the study design can be seen in Figure 2.

The total dataset comprises 899 patients who underwent surgical treatment for femoral neck fractures. The median age of the patients is 53 years (range: 42–59), with 474 males (53%) and 425 females (47%). Among these, 583 patients (65%) had non-displaced fractures (Garden I, Garden II), and 316 patients (35%) had displaced fractures (Garden III, Garden IV). Open reduction was performed in 125 cases (14%), and closed reduction in 774 cases (86%). The average surgical time was 1 h (range: 0.83–1.38), with an average intraoperative blood loss of 30 milliliters (range: 20–50). The average preoperative waiting time was 3 days (range: 2–4). All patients were followed up for at least 3 months, with the follow-up period ranging from 3 to 60 months. Additional demographic characteristics can be found in Supplementary Table S1.

Among patients who underwent surgical treatment for femoral neck fractures, 366 (41%) were fixed with FNS (Femoral Neck System), 138 (15%) with FNS + ARS (Femoral Neck System combined with Anti-Rotation Screw), 78 (8.7%) with FNS + MSP (Femoral Neck System combined with Medial Support Plate), and 317 (35%) with CCS (Cannulated Compression Screws). Of these, 68 (18.6%) FNS patients, 21 (15.2%) FNS + ARS patients, 6 (7.7%) FNS + MSP patients, and 63 (19.9%) CCS patients experienced postoperative complications, as detailed in Supplementary Table S2. In total, 158 (17.6%) patients experienced postoperative complications, while 741 (82.4%) achieved clinical cure. The statistical results show that the incidence of severe femoral neck shortening (>10 mm) was the highest, at 6.7% (*n* = 60), followed by femoral head necrosis at 5.0% (*n* = 45), which were the two major complications following FNS surgery for femoral neck fractures. Other complications had relatively low incidence rates, including nonunion at 2.2% (*n* = 19), infection at 1.1% (*n* = 10), femoral neck fixation device complications at 1.4% (*n* = 13), periprosthetic fracture at 0.6% (*n* = 5), poor wound healing at 0.4% (*n* = 4), and heterotopic ossification at the lowest rate of 0.2% (*n* = 2). The data are detailed in Table 1.

**Table 1:** Postoperative complication.

Complication	*n*, (%)
Femoral head necrosis, *n* (%)	45 (5.0%)
Nonunion, *n* (%)	19 (2.2%)
Femoral neck severe shortening, *n* (%)	60 (6.7%)
Infection, *n* (%)	10 (1.1%)
Heterotopic ossification, *n* (%)	2 (0.2%)
internal fixation failure, *n* (%)	13 (1.4%)
Peri-fixation fractures, *n* (%)	5 (0.6%)
Poor wound healing, *n* (%)	4 (0.4%)

### Screening of characteristic factors for risk

**Figure 3:**
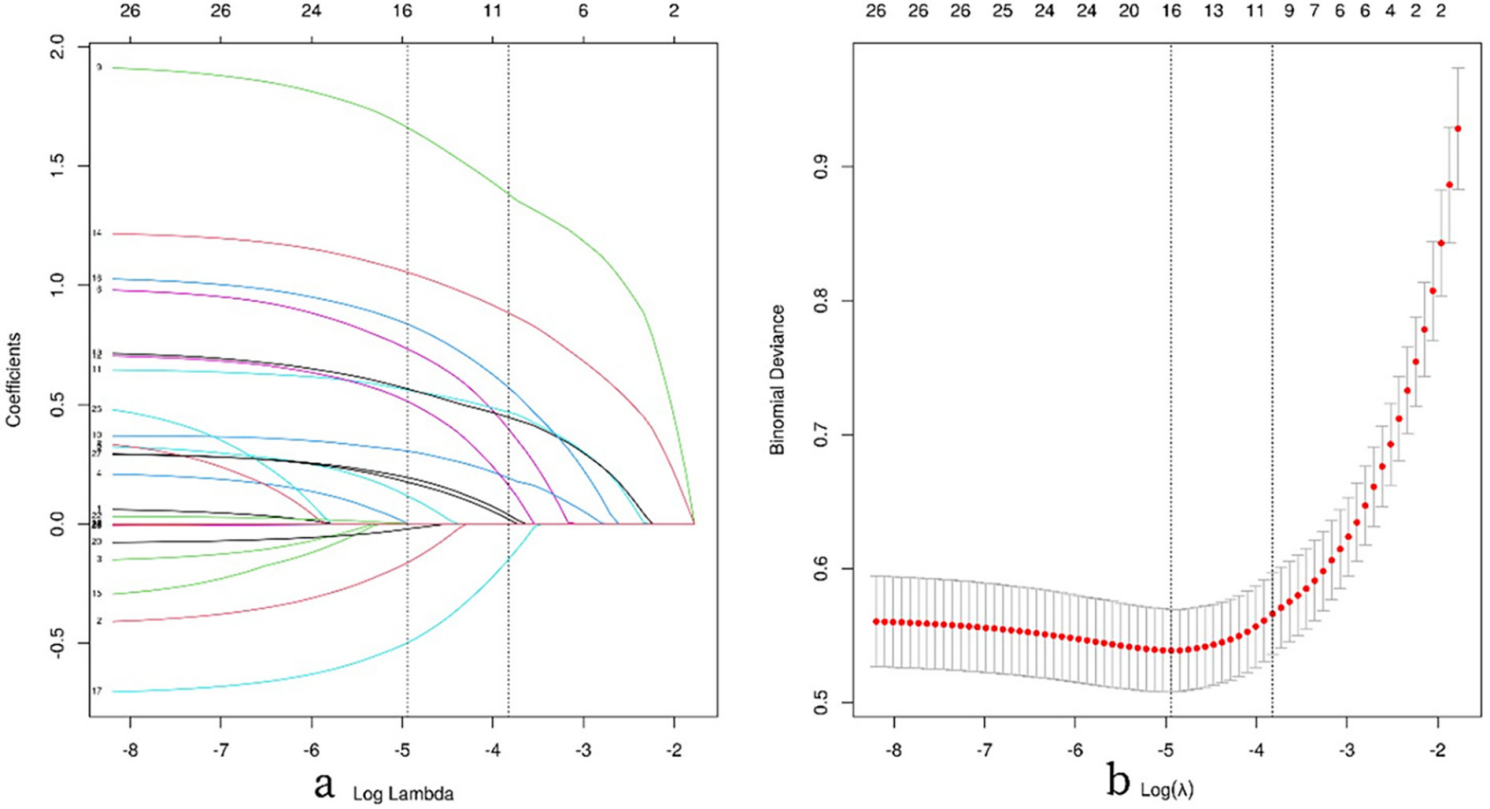
Illustrates the process of feature selection using LASSO regression analysis. **(a)** The 10-fold cross-validation results are shown, with vertical lines marking the selected values corresponding to the optimal lambda values that yield nine non-zero coefficients. **(b)** The coefficient contour plot for 42 texture features in the LASSO model is displayed, with the log(*λ*) sequence on the *x*-axis. Vertical dashed lines indicate the optimal lambda values: the minimum mean squared error (*λ* = 0.007) and the minimum standard error of the mean (*λ* = 0.022).

The results indicate that with a minimum mean squared error (*λ*) of 0.007, the 28 independent variables were reduced to 16. These include the injured side, smoking history, Charlson Comorbidity Index (CCI_score), removal of internal fixation devices, surgical approach, medial cortex comminution, Garden classification, Pauwels classification (two different fracture type classifications), injury mechanism, reduction quality (Gotfried), reduction quality (Garden index), posterior tilt angle, early postoperative weight-bearing, white blood cell count (WBC), D-dimer (D_dimer), and surgical time, as shown in Figure 3.

To further control for the influence of confounding factors, a multivariate logistic regression analysis was conducted on the aforementioned 16 independent variables. Ultimately, only the removal of fracture internal fixation devices [OR (95% CI) = 2.37 (1.22–4.57), *p* = 0.010], medial cortex comminution [OR (95% CI) = 6.19 (3.15–12.17), *p* < 0.001], fracture classification (Garden) [OR (95% CI) = 1.53 (0.78–3.01), *p* = 0.035], reduction quality (Garden index) [OR (95% CI) = 7.65 (3.05–19.24), *p* < 0.001], posterior tilt angle [OR (95% CI) = 2.66 (1.50–4.70), *p* < 0.001], and early weight-bearing [OR (95% CI) = 0.45 (0.24–0.84), *p* = 0.012] were identified as characteristic factors (*p* < 0.05), as shown in Table 2.

**Table 2:** Multifactor logistic analysis.

Variables	*β*	S.E	*Z*	*P*	OR (95%CI)
Intercept	−3.71	0.82	−4.51	<.001	0.02 (0.00–0.12)
Removal of internal fixation					
No					1.00 (Reference)
Yes	0.86	0.34	2.56	0.010	2.37 (1.22–4.57)
Medial cortical comminution					
No					1.00 (Reference)
Yes	1.82	0.34	5.29	<.001	6.19 (3.15–12.17)
Fracture type (Garden)					
Non-displaced					1.00 (Reference)
Displaced	0.42	0.35	1.23	0.035	1.53 (0.78–3.01)
Fracture type (Pauwels)					
<30°					1.00 (Reference)
30°–50°	−1.85	0.46	−4.01	<.001	0.16 (0.06–0.39)
>50°	0.24	0.44	0.54	0.590	1.27 (0.53–3.02)
Injury mechanism					
Low energy					1.00 (Reference)
High energy	0.58	0.35	1.66	0.097	1.78 (0.90–3.51)
Reduction quality (gofried)					
Optimistic					1.00 (Reference)
Satisfaction	−0.53	0.49	−1.07	0.284	0.59 (0.22–1.55)
Poor	0.92	0.53	1.74	0.082	2.50 (0.89–7.01)
Reduction quality (garden index)					
I					1.00 (Reference)
II	−0.66	0.49	−1.35	0.176	0.52 (0.20–1.35)
III	2.04	0.47	4.33	<.001	7.65 (3.05–19.24)
IV	2.26	0.52	4.36	<.001	9.60 (3.47–26.55)
Posterior tilt					
<20°					1.00 (Reference)
≥20°	0.98	0.29	3.36	<.001	2.66 (1.50–4.70)
Early postoperative weight-bearing					
No					1.00 (Reference)
Yes	−0.80	0.32	−2.51	0.012	0.45 (0.24–0.84)
Surgical time	0.30	0.20	1.54	0.123	1.35 (0.92–1.98)

OR, odds ratio; CI, confidence interval.

### Multi-model comprehensive analysis

**Figure 4:**
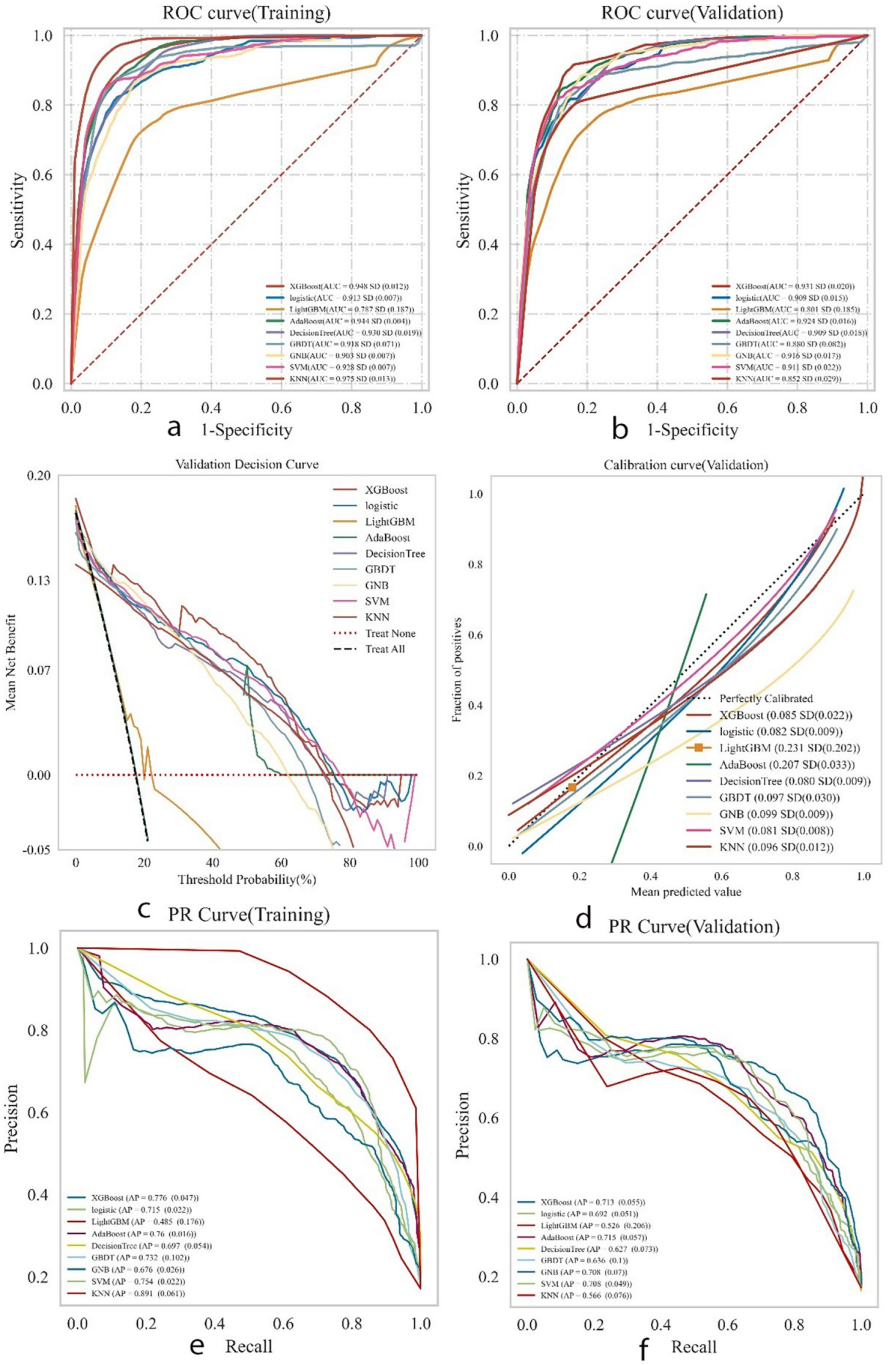
Presents the comprehensive analysis results of the machine learning models. **(a)** Displays the receiver operating characteristic (ROC) curves and area under the curve (AUC) for the training set to evaluate the models’ discrimination ability. **(b)** Shows the ROC and AUC for the test set for independent performance validation. **(c)** Presents the decision curve analysis (DCA) for the test set, where the black dashed line represents the scenario assuming all patients have postoperative complications, the red dashed line and thin black line represent the scenario assuming no complications, and the solid lines represent the performance of different models. **(d)** Illustrates the calibration curves for the test set, with the horizontal axis representing the average predicted probability by the model and the vertical axis representing the actual probability of occurrence. The dashed diagonal line indicates perfect prediction, and the smooth solid lines indicate the fit of each model. The closer the fitting line is to the diagonal, the higher the model's predictive accuracy. **(e,f)** Display the precision-recall (PR) curves and average precision (AP) for the training and test sets, respectively. In the PR curves, if one model's curve completely covers another's, it indicates superior performance of the former. Higher AP values indicate better model performance. Different colors in the figure represent different models, with values expressed as the mean and 95% confidence interval (CI).

XGBoost, Logistic Regression, LightGBM, Decision Tree, AdaBoost, GBDT, SVM, KNN, and GNB models were trained and evaluated, each repeated 10 times. The performance of these models was assessed using the area under the curve (AUC). Among the nine models, KNN achieved the highest AUC (0.975) in the training set, indicating the best predictive performance for postoperative complications, followed by XGBoost (AUC = 0.948) and AdaBoost (AUC = 0.944). Detailed performance metrics are provided in Supplementary Table S3. While KNN showed the best performance on the training set (AUC = 0.975), XGBoost achieved the highest AUC in the validation set (0.931), as shown in Figures 4a,b. KNN's performance dropped in the validation set (AUC = 0.852), suggesting a high likelihood of overfitting.In predicting postoperative complications in young and middle-aged patients with femoral neck fractures, the changes in AUC (△AUC) between training and validation sets were as follows: XGBoost △AUC = 0.017, Logistic △AUC = 0.004, LightGBM △AUC = 0.014, AdaBoost △AUC = 0.024, Decision Tree △AUC = 0.021, GBDT △AUC = 0.038, GNB △AUC = 0.013, SVM △AUC = 0.017, and KNN △AUC = 0.123. The Logistic Regression model demonstrated the best stability.Decision curve analysis (DCA) highlighted the high clinical utility of both the Logistic and XGBoost models (Figure 4C). Calibration curves showed greater predictive accuracy for the SVM and Logistic models (Figure 4D). Precision-recall (PR) curves for the training set showed average precision (AP) values of KNN = 0.891 (0.061), XGBoost = 0.776 (0.047), and Logistic = 0.715 (0.022). For the validation set, the AP values were KNN = 0.566 (0.076), XGBoost = 0.713 (0.055), and Logistic = 0.692 (0.022) (Figures 4e,f). Overall, the combined analysis suggests that the Logistic Regression model is the optimal choice.

### Final model

**Figure 5:**
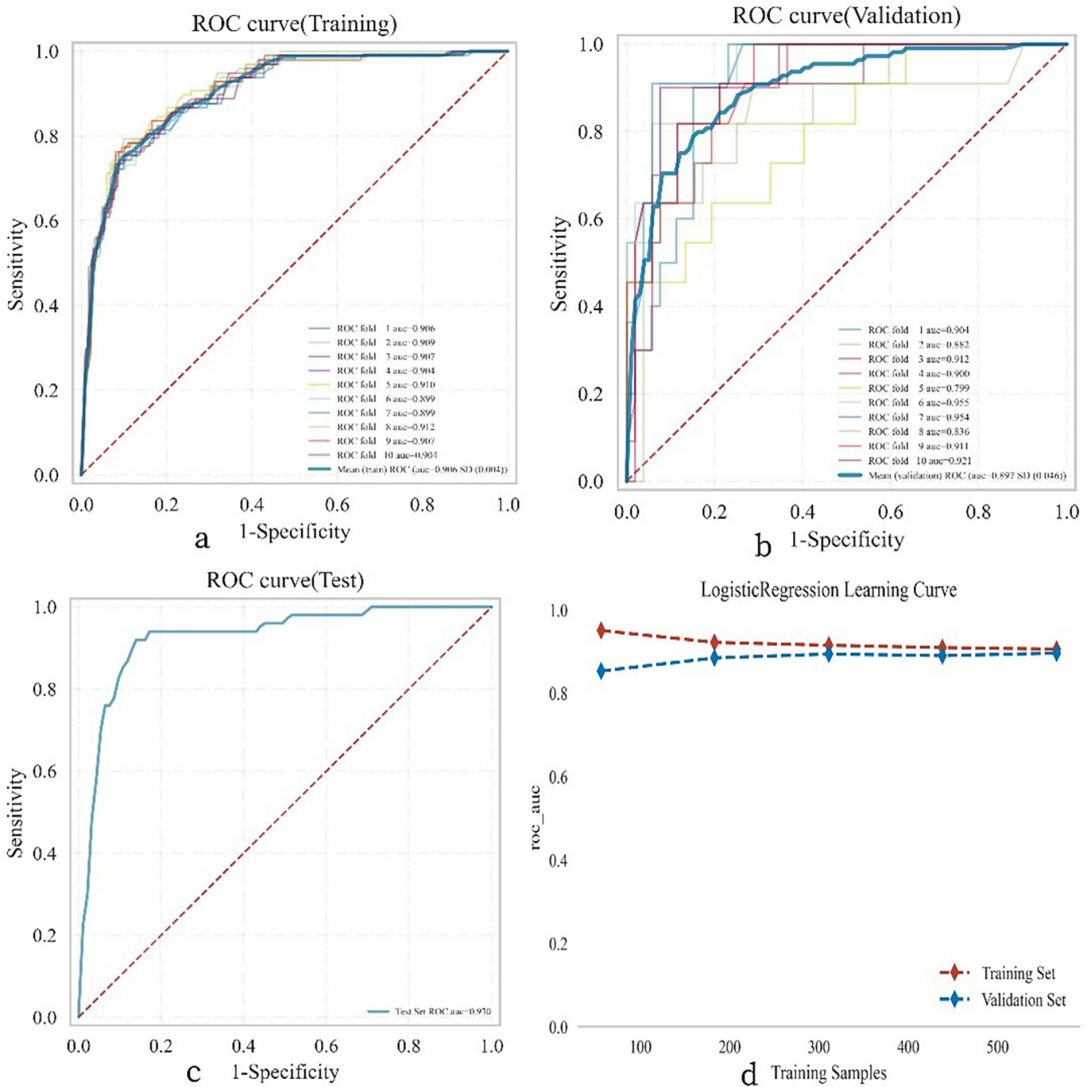
Illustrates the performance of the logistic regression model across the training, validation, and testing phases. **(a)** Displays the receiver operating characteristic (ROC) curve and the area under the curve (AUC) for the training set, with different colored solid lines representing 10 distinct training outcomes. **(b)** Shows the ROC and AUC for the validation set, which involves training and cross-validation on 10% of the femoral neck fracture postoperative patient data. **(c)** Presents the ROC and AUC for the test set, which includes data from 30% of the femoral neck fracture postoperative patients. **(d)** Depicts the learning curve, with the red dashed line representing the performance of the training set and the blue dashed line representing the performance of the validation set. The values in the figure are expressed as the mean and 95% confidence interval (CI).

A test set comprising 269 cases (30.00%) was randomly selected from the overall sample, while the remaining cases were designated as the training set for logistic regression analysis and 10-fold cross-validation. The results demonstrated (Figure 5) that the mean AUC of the training set was 0.906 (±0.004), with a sensitivity of 0.748, specificity of 0.903, positive predictive value (PPV) of 0.619, negative predictive value (NPV) of 0.945, accuracy of 0.877, and an F1 score of 0.676. The mean AUC of the validation set was 0.892 (±0.046), and that of the test set was 0.930 (±0.046). Ultimately, the AUC values of the training, validation, and test sets stabilized at 0.909, indicating accurate model predictions. Given that the AUC of the validation set is lower than that of the test set but the difference is less than 10%, the model can be deemed successfully fitted. The learning curves also suggest a strong and stable fit between the training and validation sets. These findings suggest that the logistic regression model is suitable for classification modeling tasks on this dataset. The model parameters for the training, validation, and test sets are presented in Tables 3–5.

**Table 3:** Summary of training set results.

AUC (SD)	Cutoff (SD)	Accuracy (SD)	Sensitivity (SD)	Specificity (SD)	Positive predictive value (SD)	Negative predictive value (SD)	F1 score (SD)
0.906 (0.004)	0.262 (0.027)	0.877 (0.011)	0.748 (0.028)	0.903 (0.017)	0.619 (0.034)	0.945 (0.005)	0.676 (0.017)

**Table 4:** Summary of validation set results.

AUC (SD)	Cutoff (SD)	Accuracy (SD)	Sensitivity (SD)	Specificity (SD)	Positive predictive value (SD)	Negative predictive value (SD)	F1 score (SD)
0.897 (0.046)	0.262 (0.027)	0.859 (0.048)	0.725 (0.139)	0.887 (0.053)	0.588 (0.110)	0.940 (0.030)	0.640 (0.096)

**Table 5:** Summary of test set results.

AUC (SD)	Cutoff (SD)	Accuracy (SD)	Sensitivity (SD)	Specificity (SD)	Positive predictive value (SD)	Negative predictive value (SD)	F1 score (SD)
0.93	0.267	0.885	0.88	0.886	0.638	0.97	0.739

### SHAP model explanation

**Figure 6:**
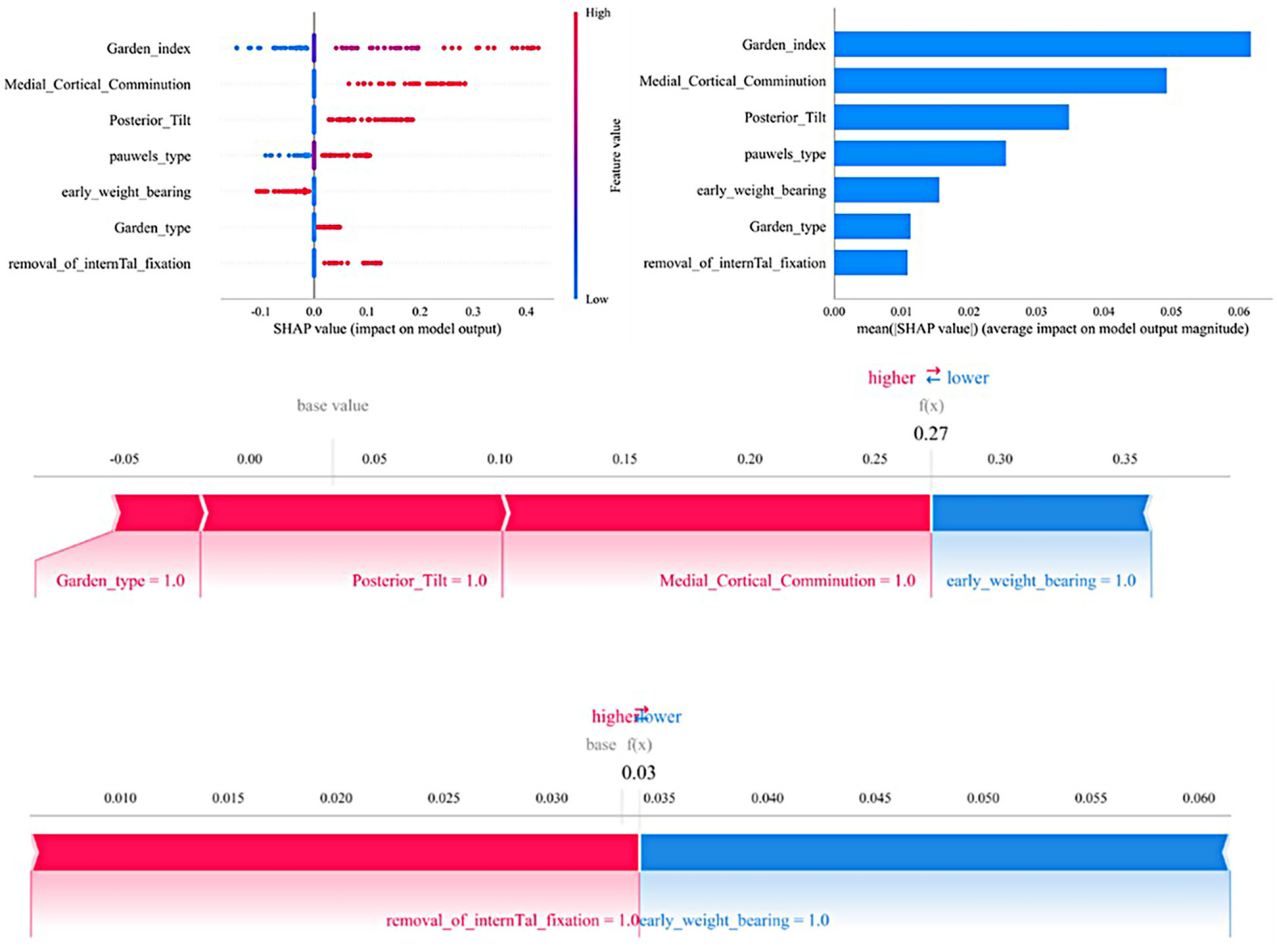
The SHAP analysis model explanation is as follows: **(a)** in the SHAP analysis, each line represents a feature, with its SHAP values displayed along the horizontal axis. Red points correspond to higher feature values, while blue points represent lower feature values. **(b)** The SHAP plot ranks the feature importance, while the matrix chart illustrates the significance of each covariate in constructing the final predictive model. **(c,d)** Display the individual contributions of patients without and with postoperative complications, respectively. The SHAP values illustrate the predictive features for each patient and their contributions to the mortality prediction. Bolded numbers indicate the predicted probability values [*f*(*x*)], with the base value representing the prediction in the absence of any input. *F*(*x*) is the log-odds ratio of the observed values. Red features indicate an increased risk of death, while blue features signify a reduced risk. The length of the arrows reflects the magnitude of the predictive impact; the longer the arrow, the greater the effect.

Figure 6 illustrates the seven most important features in our model. These features include removal of the fracture internal fixation device, medial cortical comminution, fracture type (Garden classification), fracture type (Pauwels classification), quality of reduction (Garden index), posterior inclination, and early weight bearing.For each characteristic, the contributions of all patients to the outcomes are plotted along significant lines, represented by differently colored dots. Red dots indicate high-risk values, while blue dots indicate low-risk values. The ranking of the seven risk factors is determined by their mean absolute SHAP (SHapley Additive exPlanations) values, with the *x*-axis representing the SHAP values that indicate the importance of each feature in the predictive model.Additionally, two typical examples are provided to demonstrate the interpretability of the model. One example is a postoperative patient with a fractured femoral neck who experienced postoperative complications and had a higher SHAP prediction score. The other example is a postoperative patient without complications, who had a lower SHAP score.

### Convenient application for clinical utility

**Figure 7:**
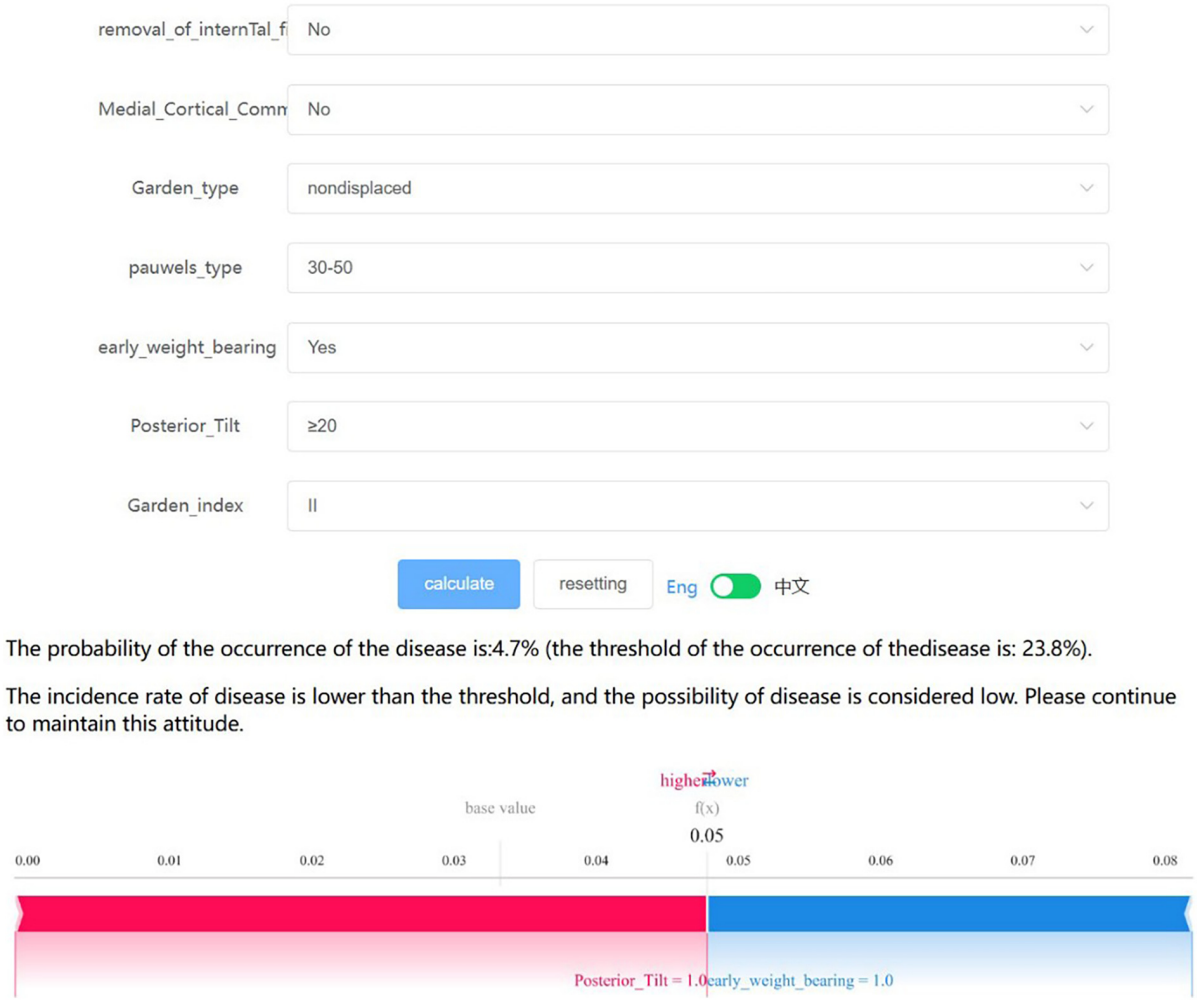
The final logistic regression model, which incorporates 7 features, facilitates the prediction of postoperative complication rates in young and middle-aged patients following femoral neck fracture surgery. Upon inputting the actual values for these 7 features, the application will automatically calculate and display a 4.7% probability of complications. Additionally, the force diagram for individual patients highlights the features contributing to the prediction of postoperative complications: features on the right side (colored in blue) indicate factors that reduce the likelihood of complications, while features on the left side (colored in red) represent factors that increase the likelihood of complications.

The final predictive model has been implemented into a web application to enhance its practicality in clinical settings, as shown in Figure 7. When the actual values of the seven required features are input, the application automatically predicts the risk of postoperative complications for individual patients. Additionally, a force diagram is displayed to highlight the features contributing to the prediction of surgical complications: features on the right side in blue indicate those that push the prediction towards “no complications,” while features on the left side in red indicate those that push the prediction towards “complications.” The web application can be accessed online at http://www.xsmartanalysis.com/model/list/predict/model/html?mid=19041&symbol=5WMNGQt17310737ZM894.

## Discussions

This study aimed to evaluate and compare nine machine learning (ML) models for predicting postoperative complications in young and middle-aged patients with femoral neck fractures. We identified key predictive risk factors and utilized machine learning algorithms, along with clinical and laboratory data, to construct a predictive model for postoperative complications in femoral neck fracture patients.

### Advantages of machine learning algorithms

Although numerous studies have focused on predicting postoperative femoral head necrosis (22, 42), the multifaceted complications associated with femoral neck fracture internal fixation make it challenging to predict a single complication in clinical practice. Machine learning (ML) is a powerful computational method capable of handling complex and extensive datasets, understanding intricate relationships between variables, and adapting through training. The integration of clinical data with advanced ML algorithms facilitates the development of robust predictive models. Of the nine ML models, the logistic regression model demonstrated the best area under the curve (AUC) value, offering optimal net benefit and a high threshold probability for feature reduction. Among the eleven machine learning (ML) models evaluated, the logistic regression model showed the highest AUC value, demonstrating superior net benefit and a high threshold probability during feature selection. Logistic regression is a commonly used classification algorithm that predicts event probabilities based on the impact of multiple variables on the outcome. In this study, we selected the logistic regression model due to its strong statistical performance and excellent interpretability. A final model incorporating seven features was developed using the logistic regression algorithm. These features can be assessed during the perioperative period and postoperative follow-up, positioning the model as a potential tool for the long-term dynamic monitoring of postoperative complication risks in young and middle-aged femoral neck fracture patients.

The lack of established guidelines or consensus regarding feature selection for predictive model construction leaves the optimal number of features uncertain. While incorporating additional features may enhance the model's predictive power, an excessive number of features could hinder its clinical applicability, and the inclusion of non-causal features may compromise predictive accuracy. The SHAP method was employed to aid in the selection of relevant features. The final model developed, a simple and user-friendly machine learning (ML) prediction tool, is expected to be readily adopted to support clinical decision-making for young and middle-aged femoral neck fracture patients.

### Risk and protective factors

The results of this study indicate that the quality of intraoperative reduction is a strong predictor of postoperative complications following femoral neck surgery, with poor reduction quality (Garden index III and IV) and medial cortex comminution being the most significant predictive factors. Furthermore, displaced fractures (Garden III and IV), Pauwels III, posterior tilt angle ≥20°, and removal of internal fixation devices were identified as key variables.

Our study indicates that poor reduction quality, as indicated by Garden index III [OR (95% CI) = 7.65 (3.05–19.24), *P* < 0.001], and Garden index IV [OR (95% CI) = 9.60 (3.47–26.55), *p* < 0.001], are significant risk factors for failure of the Femoral Neck System (FNS). Several studies have demonstrated that poor reduction can lead to fracture re-displacement, thereby increasing the risk of internal fixation failure (15, 43). A study by Zhang Jin et al. (44) further confirmed that poor reduction quality is a significant risk factor for the development of femoral head necrosis. Therefore, during surgical planning and execution, it is critical to ensure precise alignment and handling of the fracture line. The pre-sliding technique is recommended to optimize screw trajectory and positioning, which has been shown to reduce the risk of femoral neck shortening (45). Notably, Li et al. (46) found, through a literature analysis, that active support and anatomic reduction based on Gotfried criteria significantly reduce postoperative complications, including ischemic necrosis of the femoral head. This finding contrasts with the results of our study, which showed that reduction quality, as assessed by positive and negative support theories, did not significantly impact the occurrence of postoperative complications.

Medial cortex comminution is a well-established major risk factor for postoperative complications following femoral neck fractures (11, 12, 47), commonly resulting from high-energy trauma, which can disrupt blood supply and consequently elevate the risk of complications. Comminuted or free bone fragments in the medial cortex of the femoral neck impair effective stress distribution and weight-bearing, disrupting the biomechanical environment and increasing the risk of complications, such as exacerbated bone resorption, deformity progression, and fixation failure. Other risk factors, including displaced fractures, a posterior tilt angle ≥20°, and removal of internal fixation devices, interact with biomechanical and biological factors, influencing the prognosis following femoral neck fracture surgery.

The results of this study indicate that early weight-bearing [OR (95% CI) = 0.45 (0.24–0.84), *P* = 0.012] serves as a protective factor against postoperative complications in young and middle-aged patients with femoral neck fractures. We recommend that patients with stable fractures (Garden I and Garden II) or those with good reduction (Garden index I and Garden index II) engage in early, limited weight-bearing. Research suggests that for fractures with a low Pauwels angle, early weight-bearing can convert compressive stress into healing forces (48). In patients with significantly displaced fractures, achieving good intraoperative reduction can reconstruct bone structure and restore stress transmission (46). Our study employed a personalized weight-bearing strategy, advising patients to begin bearing a tolerable load of 20–30 kilograms within three weeks postoperatively, gradually increasing the load according to radiological healing progress until full weight-bearing is achieved. In comparison with the guidelines by Kubiak et al. (49),which recommend that femoral neck fracture patients under the age of 65 begin weight-bearing exercises between six and twelve weeks postoperatively, our approach advocates for earlier weight-bearing. Therefore, we emphasize the importance of an early and progressive weight-bearing regimen to optimize fracture healing and mitigate the risk of postoperative complications. Early postoperative weight-bearing management can minimize adverse effects at the fracture site, apply compressive stress to facilitate healing, and enhance fracture consolidation quality.

This study shows that the removal of internal fixation devices is an independent characteristic factor. This suggests that in clinical practice, extra caution should be exercised when considering the removal of internal fixation devices, especially when fracture healing is incomplete or other potential risk factors are present. The removal of internal fixation devices may disrupt the biomechanical environment during the fracture healing process, leading to redisplacement or instability at the fracture site and consequently affecting the blood supply to the femoral head. Additionally, the presence of internal fixation devices may offer some protective effect on the surrounding tissues, and the loss of this protection following removal could render the femoral head more vulnerable to injury.

### Preoperative waiting time

When evaluating the impact of preoperative waiting time on postoperative complications in femoral neck fractures, it is essential to consider the existing body of research. A meta-analysis by Costas Papakostidis et al. (50) concluded that surgical timing is not a risk factor for postoperative complications in femoral neck fractures, which challenges the traditional belief that early surgery reduces postoperative complications. Another study (51) also highlighted that, although timely surgery may reduce the ischemic time of the femoral head, performing surgery within 24 h of injury does not significantly decrease the incidence of femoral head necrosis compared to delayed surgery. This suggests that, in addition to surgical timing, factors such as surgical technique, methods of internal fixation, and individual patient differences may play a more significant role in the occurrence of postoperative complications in femoral neck fractures.

### Significance of model development

The machine learning prediction model developed in this study holds great significance. On one hand, it integrates various factors, including patient age, fracture type, surgical procedure, and preoperative and postoperative metrics, to provide accurate risk predictions for each patient. This enables the identification of high-risk patients and the development of preemptive interventions. The model is not only applicable for preoperative assessment but also for dynamic monitoring of complication risks during postoperative follow-up, facilitating early detection and intervention of potential issues. On the other hand, it aids in optimizing clinical decision-making and enhancing treatment outcomes. Accurate preoperative risk prediction assists surgeons in selecting the most suitable surgical options for patients, such as carefully choosing the timing or modality of surgery. Additionally, the model can inform rehabilitation planning based on postoperative complication predictions, allocate medical resources rationally, improve resource utilization efficiency, reduce additional medical costs resulting from complications, and alleviate patient pain and medical burdens.

### Limitations

This study has several limitations. First, the data for this study were collected from a single institution rather than through a multicenter approach. Consequently, the generalizability of the results may be limited. Second, although high consistency was achieved in the repeatability analysis within the training and testing sets, some inevitable errors could arise due to the inherent uncertainty in the data split. Lastly, the study design did not include certain variables in the analysis, such as the anteversion angle and bone density. Longitudinal or prospective case-control studies are needed to further elucidate the relationship between risk factors and the development of postoperative complications in femoral neck fractures.

## Conclusion

In summary, this study developed a predictive model using machine learning (ML) algorithms, with the Logistic regression model showing superior performance. Additionally, we provided a personalized risk assessment for postoperative complications in young and middle-aged femoral neck fracture patients using SHAP explanations. This computer-assisted method can effectively help clinicians and patients dynamically monitor the risk of postoperative complications.

## Data Availability

The original contributions presented in the study are included in the article/Supplementary Material, further inquiries can be directed to the corresponding author/s.
