# Supplementary material for: A machine learning model for predicting postoperative complication risk in young and middle-aged patients with femoral neck fractures
Source: Front Surg. 2025 Aug 26;12:1591671. doi: 10.3389/fsurg.2025.1591671 (PMC12417505; doi:10.3389/fsurg.2025.1591671)
Supplement: Supplementary file 1 [file Table1.docx]

S 1 Baseline Table

|  | group | | |  |
| --- | --- | --- | --- | --- |
| Variable | Overall, N = 899^1^ | No Complications, N = 741^1^ | With Complications, N = 158^1^ | p-value^2^ |
| Age, Median (IQR) | 53.000 (42.000 – 59.000) | 53.000 (42.000 – 59.000) | 52.500 (43.000 – 57.000) | 0.271 |
| BMI, Median (IQR) | 21.778 (18.508 – 23.671) | 21.830 (18.645 – 23.613) | 21.221 (17.537 – 23.702) | 0.424 |
| Surgical_time, Median (IQR) | 1.000 (0.830 – 1.380) | 1.000 (0.780 – 1.330) | 1.160 (0.913 – 1.443) | <0.001 |
| Preoperative_waiting_time, Median (IQR) | 3.000 (2.000 – 4.000) | 3.000 (2.000 – 4.000) | 3.000 (2.000 – 5.000) | 0.620 |
| intraoperative_blood_loss, Median (IQR) | 30.000 (20.000 – 50.000) | 30.000 (20.000 – 50.000) | 30.000 (20.000 – 50.000) | 0.060 |
| Hb, Median (IQR) | 9.920 (8.080 – 11.530) | 9.920 (8.080 – 11.530) | 9.575 (7.850 – 11.473) | 0.252 |
| WBC, Median (IQR) | 9.100 (7.600 – 10.900) | 9.100 (7.500 – 10.800) | 9.100 (7.700 – 11.100) | 0.634 |
| PLT, Median (IQR) | 273.000 (203.000 – 310.000) | 273.000 (204.000 – 310.000) | 274.000 (201.000 – 306.000) | 0.889 |
| D_dimer, Median (IQR) | 5.700 (1.590 – 11.158) | 5.630 (1.590 – 11.080) | 6.391 (1.575 – 11.868) | 0.604 |
| Gender, n (%) |  |  |  | 0.517 |
| Female | 425 (47) | 354 (48) | 71 (45) |  |
| Male | 474 (53) | 387 (52) | 87 (55) |  |
| Side, n (%) |  |  |  | 0.777 |
| Left | 446 (50) | 366 (49) | 80 (51) |  |
| Right | 453 (50) | 375 (51) | 78 (49) |  |
| Alcoholist, n (%) |  |  |  | 0.042 |
| No | 469 (52) | 375 (51) | 94 (59) |  |
| Yes | 430 (48) | 366 (49) | 64 (41) |  |
| smoking, n (%) |  |  |  | 0.691 |
| No | 632 (70) | 523 (71) | 109 (69) |  |
| Yes | 267 (30) | 218 (29) | 49 (31) |  |
| CCI_score, n (%) |  |  |  | 0.335 |
| 0 points | 730 (81) | 606 (82) | 124 (78) |  |
| ≥1 points | 169 (19) | 135 (18) | 34 (22) |  |
| removal_of_internTal_fixation, n (%) |  |  |  | <0.001 |
| No | 750 (83) | 637 (86) | 113 (72) |  |
| Yes | 149 (17) | 104 (14) | 45 (28) |  |
| surgical procedure, n (%) |  |  |  | 0.066 |
| FNS | 366 (41) | 298 (40) | 68 (43) |  |
| FNS+ARS | 138 (15) | 117 (16) | 21 (13) |  |
| FNS+MSP | 78 (8.7) | 72 (9.7) | 6 (3.8) |  |
| CCS | 317 (35) | 254 (34) | 63 (40) |  |
| Intertrochanteric Fracture, n (%) |  |  |  | 0.962 |
| No | 870 (97) | 717 (97) | 153 (97) |  |
| Yes | 29 (3.2) | 24 (3.2) | 5 (3.2) |  |
| Medial Cortical Comminution, n (%) |  |  |  | <0.001 |
| No | 711 (79) | 648 (87) | 63 (40) |  |
| Yes | 188 (21) | 93 (13) | 95 (60) |  |
| fracture_type (Garden), n (%) |  |  |  | <0.001 |
| Non-displaced | 583 (65) | 535 (72) | 48 (30) |  |
| Displaced | 316 (35) | 206 (28) | 110 (70) |  |
| fracture_type (Pauwels), n (%) |  |  |  | <0.001 |
| ＜30° | 168 (19) | 146 (20) | 22 (14) |  |
| 30°-50° | 497 (55) | 473 (64) | 24 (15) |  |
| ＞50° | 234 (26) | 122 (16) | 112 (71) |  |
| injury_mechanism, n (%) |  |  |  | 0.003 |
| Low Energy | 734 (82) | 618 (83) | 116 (73) |  |
| High Energy | 165 (18) | 123 (17) | 42 (27) |  |
| Reduction_quality (gofried), n (%) |  |  |  | <0.001 |
| Optimistic | 137 (15) | 124 (17) | 13 (8.2) |  |
| Satisfaction | 594 (66) | 543 (73) | 51 (32) |  |
| Poor | 168 (19) | 74 (10.0) | 94 (59) |  |
| Reduction_quality (garden index), n (%) |  |  |  | <0.001 |
| I | 161 (18) | 146 (20) | 15 (9.5) |  |
| II | 486 (54) | 467 (63) | 19 (12) |  |
| III | 164 (18) | 90 (12) | 74 (47) |  |
| IV | 88 (9.8) | 38 (5.1) | 50 (32) |  |
| reduction_methor, n (%) |  |  |  | 0.618 |
| Closed | 774 (86) | 636 (86) | 138 (87) |  |
| Open | 125 (14) | 105 (14) | 20 (13) |  |
| Posterior_Tilt, n (%) |  |  |  | <0.001 |
| ＜20° | 610 (68) | 543 (73) | 67 (42) |  |
| ≥20° | 289 (32) | 198 (27) | 91 (58) |  |
| Final_postoperative_Harrisi |  |  |  | <0.001 |
| ≥70 | 685（76） | 658（92） | 27（14） |  |
| ＜70 | 214（24） | 83（8） | 131（86） |  |
| early postoperative weight-bearing, n (%) |  |  |  | <0.001 |
| No | 569 (63) | 449 (61) | 120 (76) |  |
| Yes | 330 (37) | 292 (39) | 38 (24) |  |
| ^1^Median (IQR) or Frequency (%) | | | | |
| ^2^Wilcoxon rank sum test; Pearson's Chi-squared test | | | | |

S2 Complications of internal fixation devices

|  | Prognosis | | |
| --- | --- | --- | --- |
| Surgical Approach | No Complications | With Complications | Total |
| FNS，n(%) | 298(81.4%) | 68(18.6%) | 366 |
| FNS+ARS，n(%) | 117(84.8%) | 21(15.2%) | 138 |
| FNS+MSP，n(%) | 72(92.3%) | 6(7.7%) | 78 |
| CCS，n(%) | 254(80.1%) | 63(19.9%) | 317 |

S3Multimodel Classification - Training Set Results Summary

| Classification Model | AUC(SD) | cutoff(SD) | Accuracy (SD) | Sensitivity (SD) | Specificity (SD) | Positive Predictive Value (SD) | Negative Predictive Value (SD) | F1 Score (SD) | Kappa(SD) |
| --- | --- | --- | --- | --- | --- | --- | --- | --- | --- |
| XGBoost | 0.948(0.012) | 0.283(0.062) | 0.886(0.014) | 0.878(0.030) | 0.888(0.019) | 0.621(0.042) | 0.972(0.007) | 0.726(0.027) | 0.657(0.034) |
| logistic | 0.913(0.007) | 0.229(0.026) | 0.871(0.011) | 0.808(0.027) | 0.885(0.016) | 0.602(0.029) | 0.956(0.006) | 0.689(0.017) | 0.610(0.023) |
| LightGBM | 0.787(0.187) | 0.148(0.077) | 0.763(0.189) | 0.545(0.365) | 0.810(0.263) | NaN(NaN) | 0.895(0.061) | NaN(NaN) | 0.298(0.257) |
| AdaBoost | 0.944(0.004) | 0.470(0.031) | 0.870(0.021) | 0.879(0.029) | 0.867(0.031) | 0.591(0.046) | 0.971(0.005) | 0.705(0.025) | 0.626(0.037) |
| DecisionTree | 0.930(0.019) | 0.248(0.066) | 0.865(0.031) | 0.792(0.062) | 0.882(0.036) | 0.605(0.082) | 0.951(0.015) | 0.682(0.057) | 0.599(0.075) |
| GBDT | 0.918(0.071) | 0.188(0.065) | 0.885(0.032) | 0.859(0.069) | 0.890(0.040) | 0.638(0.070) | 0.968(0.014) | 0.729(0.056) | 0.659(0.074) |
| GNB | 0.903(0.007) | 0.183(0.070) | 0.833(0.012) | 0.855(0.026) | 0.828(0.019) | 0.518(0.027) | 0.964(0.006) | 0.645(0.015) | 0.544(0.020) |
| SVM | 0.928(0.007) | 0.150(0.029) | 0.896(0.009) | 0.847(0.026) | 0.907(0.015) | 0.664(0.033) | 0.965(0.006) | 0.743(0.015) | 0.680(0.020) |
| KNN | 0.975(0.013) | 0.223(0.034) | 0.929(0.014) | 0.907(0.050) | 0.933(0.012) | 0.746(0.041) | 0.979(0.011) | 0.818(0.038) | 0.774(0.046) |

S4 Multimodel Classification - Validation Set Results Summary

| Classification Model | AUC(SD) | cutoff(SD) | Accuracy (SD) | Sensitivity (SD) | Specificity (SD) | Positive Predictive Value (SD) | Negative Predictive Value (SD) | F1 Score (SD) | Kappa(SD) |
| --- | --- | --- | --- | --- | --- | --- | --- | --- | --- |
| XGBoost | 0.931(0.020) | 0.283(0.062) | 0.874(0.025) | 0.839(0.059) | 0.883(0.028) | 0.622(0.056) | 0.960(0.018) | 0.712(0.041) | 0.634(0.056) |
| logistic | 0.909(0.015) | 0.229(0.026) | 0.861(0.018) | 0.786(0.045) | 0.878(0.020) | 0.577(0.047) | 0.951(0.012) | 0.664(0.035) | 0.579(0.045) |
| LightGBM | 0.801(0.185) | 0.148(0.077) | 0.769(0.190) | 0.553(0.374) | 0.813(0.268) | NaN(NaN) | 0.893(0.059) | NaN(NaN) | 0.323(0.294) |
| AdaBoost | 0.924(0.016) | 0.470(0.031) | 0.857(0.033) | 0.829(0.040) | 0.863(0.042) | 0.572(0.072) | 0.959(0.010) | 0.674(0.050) | 0.587(0.067) |
| DecisionTree | 0.909(0.018) | 0.248(0.066) | 0.854(0.036) | 0.748(0.079) | 0.873(0.042) | 0.548(0.091) | 0.947(0.014) | 0.629(0.077) | 0.541(0.094) |
| GBDT | 0.880(0.082) | 0.188(0.065) | 0.850(0.019) | 0.811(0.082) | 0.860(0.033) | 0.549(0.075) | 0.955(0.022) | 0.648(0.044) | 0.558(0.053) |
| GNB | 0.916(0.017) | 0.183(0.070) | 0.834(0.014) | 0.852(0.077) | 0.831(0.023) | 0.513(0.039) | 0.965(0.017) | 0.638(0.038) | 0.539(0.041) |
| SVM | 0.911(0.022) | 0.150(0.029) | 0.868(0.019) | 0.815(0.059) | 0.879(0.029) | 0.588(0.050) | 0.958(0.013) | 0.680(0.032) | 0.600(0.040) |
| KNN | 0.852(0.029) | 0.223(0.034) | 0.869(0.019) | 0.719(0.089) | 0.898(0.027) | 0.599(0.057) | 0.941(0.013) | 0.651(0.057) | 0.571(0.060) |
